# Dendrimer antibody conjugate to target and image HER-2 overexpressing cancer cells

**DOI:** 10.18632/oncotarget.9081

**Published:** 2016-04-28

**Authors:** James B. Otis, Hong Zong, Alina Kotylar, Anna Yin, Somnath Bhattacharjee, Han Wang, Baker James R., Su He Wang

**Affiliations:** ^1^ Department of Internal Medicine, Division of Allergy, Michigan Nanotechnology Institute for Medicine and Biological Sciences, University of Michigan, Ann Arbor, Michigan, USA; ^2^ Department of Radiology, Shanghai General Hospital, Shanghai Jiao Tong University School of Medicine, Shangai, P.R.China

**Keywords:** anti-HER2-monoclonal antibody, gold nanoparticles, PAMAM dendrimer, imaging agent, targeting

## Abstract

Although many breast and lung cancers overexpress human epidermal growth factor receptor-2 (HER-2), no methods currently exist for effective and early detection of HER-2-positive cancers. To address this issue, we designed and synthesized dendrimer-based novel nano-imaging agents that contain gold nanoparticles (AuNPs) and gadolinium (Gd), conjugated with the humanized anti-HER-2 antibody (Herceptin). Generation 5 (G5) polyamidoamine (PAMAM) dendrimers were selected as the backbone for the nano-imaging agents due to their unique size, high ratio of surface functional groups and bio-functionality. We modified G5 PAMAM dendrimer surface with PEG and 1,4,7,10-tetraazacyclododecane-1,4,7,10-tetraacetic acid (DOTA) chelators to encapsulate AuNPs and complex Gd. These dendrimer entrapped AuNPs were further conjugated with Herceptin through copper-catalyzed azide- alkyne click reaction to construct the nano-imaging agent Au-G5-Gd-Herceptin. The targeted nano-imaging agent bound selectively to HER-2 overexpressing cell lines, with subsequent internalization into the cells. More importantly, non-targeted nano-imaging agent neither bound nor internalized into cells overexpressing HER-2. These results suggest that our approach could provide a platform to develop nano-diagnostic agents or nano-therapeutic agents for early detection and treatment of HER-2-positive cancers.

## INTRODUCTION

Nanotechnology has great potential for the development of imaging agents and delivery systems of biologically active compounds [1]. Dendrimers as nanoscale particles have been developed as multifunctional vehicles as they possess a three-dimensional architecture with high density of peripheral functional groups. Additionally, the sizes and polydispersity indexes (PDI) of the dendrimers can easily be controlled [2, 3] and they are biocompatible [4]. Therefore, dendrimers are attractive macromolecules for drug delivery and molecular imaging [5, 6]. In particular, polyamidoamine (PAMAM) dendrimers have a narrow polydispersity and contain terminal amines that can be conjugated with targeting agents, therapeutics, and imaging agents for drug delivery and *in vitro* imaging [7–9].

Computerized tomography (CT) and magnetic resonance imaging (MRI) are effective techniques employed in early cancer diagnosis because of CT's high spatial and density resolution and MRI's effective resolution of soft tissues [10–12]. The capabilities of CT and MRI are dependent on the characteristics of their respective contrast agents such as length of circulation time, biocompatibility, and x-ray attenuation or relaxivity [13, 14]. The use of CT in particular has been improved using contrast agents better than popular iodine based ionic and non-ionic agents [15–17]. These improvements to CT stem in part from gold nanoparticles (AuNPs), which offer increased resolution and contrast because of their higher X-ray attenuation factor [18, 19]. Furthermore, AuNPs in a variety of instances, including encapsulation within dendrimers, have also been shown to possess heightened biocompatibility and stability [15, 20, 21]. With regard to MRI, Gadolinium (Gd) chelated into 1,4,7,10-tetraazacyclododecane-1,4,7,10-tetraacetic acid (DOTA) is a long recognized contrast agent because of its unique soft tissue resolving capabilities [22, 23]. When DOTA-Gd was conjugated to dendrimers, the relaxivity and retention time of the contrast agent was increased [24]. Moreover, targeting of these compounds using small molecules such as folate or RGD peptide against folate receptor or integrin is also possible [25, 26].

In this study, we first developed a method to functionalize G5 PAMAM dendrimers with anti HER-2-monoclonal antibody (Herceptin), DOTA-Gd, and Alexa Fluor 647 (AF647) dye, as well as entrap AuNPs to prepare the multifunctional conjugate Au-G5-Gd-Herceptin-AF647, **7**. Next, we evaluated the ability of **7** to bind and internalize into cell lines overexpressing HER-2, which to the best of our knowledge has not been previously demonstrated. HER-2 of the human epidermal growth factor receptor family was selected because HER-2 is often overexpressed in certain types of epithelia cancer including breast cancer and lung cancer [27, 28]. Indeed, the anti-HER-2 antibody has been utilized for cancer therapy as well as employed within the scope of targeted cancer cells and drug delivery [29–31].

Before conjugation with Herceptin, the G5 PAMAM dendrimer surface amino groups were conjugated with monomethyl polyethylene glycol (PEG), alkyne linkers, DOTA, and finally capped with acetylation. These modification steps extend the circulation half-life of conjugates in the reticuloendothelial system, enable the addition of functional groups via click chemistry, and reduce the non-specific binding and cytotoxicity [4, 32]. AuNPs and Gd were entrapped within G5 dendrimers and DOTA respectively to create a CT and MRI agent. We then attach the G5 dendrimer to Herceptin, using highly efficient and specific copper(I)-catalyzed alkyne-azide cycloaddition reaction (CuAAC). Finally, AF647 was conjugated to the G5 dendrimer for *in vitro* binding efficacy studies. Our results indicate that the Herceptin dendrimer conjugate **7** can serve as a targeted nano-platform for multimodal imaging agents.

## RESULTS AND DISCUSSION

### Synthesis of Au-G5-Gd-Herceptin-AF647

**Scheme 1:**
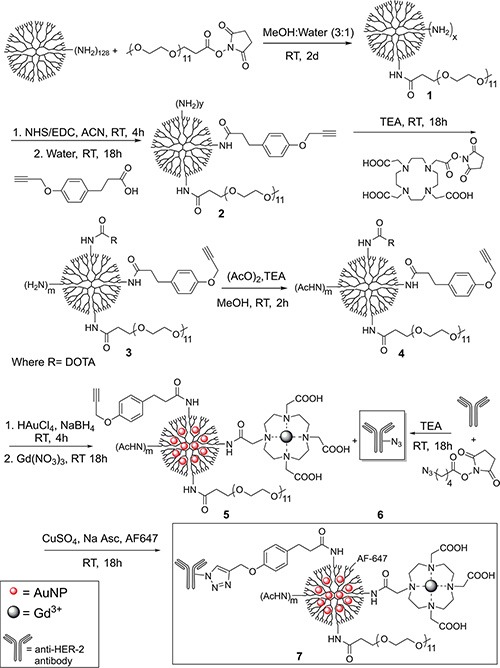
Schematic illustration of preparation of conjugate 7

Readily modifiable, a narrow polydispersity index, and proven functionality *in vitro* and *in vivo* make dendrimers well suited for targeted drug delivery and imaging applications. In contrast, utilizing Au and Gd dendrimer-entrapped nanoparticles (DENPs) requires specific targeting agents. In an effort to broaden the available targeting moieties for Au-Gd DENPs, we prepared the Herceptin dendrimer complex 7 in six steps as illustrated in Scheme 1. First, PEG was coupled to the terminal amines of G5 PAMAM dendrimers to yield G5-PEG-NH_2_ 1. Previous reports indicated that PEGylation extends dendrimer circulation half-life, and expands the dendrimer's periphery to enhance AuNPs loading [14]. G5-PEG-NH_2_ 1 was analyzed by ^1^H NMR and MALDI-TOF. ^1^H NMR showed broad peaks from 3.61 to 3.81 ppm that correspond to −CH_2_CH_2_O and −OCH_3_ of PEGs (Figure S1). We estimated from ^1^H NMR, following the method developed by Majors et al., [32] that an average of about 23 PEG molecules were attached to each dendrimer. MALDI-TOF showed higher mass of 1 than unmodified G5 (Table 1, Figure S2). The molecular weight (MW) obtained from MALDI indicated that compound 1 has 22.8 PEG moieties, which is in agreement with the results obtained from ^1^H NMR (Figure S1).

**Table 1:** Yield of each conjugate step and the respective average molecular weight

Conjugates	M.W.	% Yield
G5-NH_2_	26,254.7	-
G5-PEG-NH_2_ (**1**)	39,385.0	87%
G5-PEG-Alkyne-NH_2_ (**2**)	42,634.5	94%
G5-PEG-Alkyne-DOTA-NH_2_ (**3**)	46,185.9	85%
G5-PEG-Alkyne-DOTA-NHAc (**4**)	49,625.7	88%
Au-G5-PEG-Alkyne-DOTA-Gd-NHAc (**5**)	53,278.2	91%
Herceptin-azide (**6**)	~150,000	39%
Au-G5-Gd-Herceptin-AF647 (**7**)	~440,000	92%

Subsequently, G5-PEG-Alkyne-NH_2_ 2 was synthesized by reacting 1 with 3-(4-(ethynyloxy)phenyl)propanoic acid (alkyne linker) in the presence of NHS and 1-Ethyl-3-(3-dimethylaminopropyl)carbodiimide (EDC) at room temperature [8, 33, 34]. Alkyne linkers on conjugate 2 were employed later in click reaction with Herceptin because click reaction offers high efficiency and specificity in conjugation and can be carried out in aqueous solutions under mild conditions [8]. Additionally, click reaction enables specific dendrimer modification subsequent to capping, circumventing potential negative alterations to targeting moieties that may otherwise occur during the capping process. As with 1,^1^H NMR and MALDI-TOF were also employed to analyze conjugate 2 after purification. MALDI-TOF indicated about 16.5 alkyne linkers on average were conjugated with each dendrimer (Table 1, Figure S2). Additionally, the two broad singlets at 6.9 and 7.2 ppm that appear in the ^1^H-NMR spectra (Figure S1) correspond to the aromatic proton, further confirming the presence of the alkyne linker [8].

Next, DOTA-NHS was coupled to 2 to synthesize G5- PEG-Alkyne-DOTA-NH_2_, 3, which was then reacted with acetic anhydride to prepare G5-PEG-AlkyneDOTA- NHAc, 4. MALDI-TOF found a higher mass of 3 than 2, which also indicated that DOTAs were successfully conjugated to G5 dendrimer. Formation of 4 was confirmed both by MALDI-TOF, showing an average of 58.6 acetyl groups per dendrimer, and by the appearance of a broad singlet at 1.99 ppm in ^1^H NMR spectra (Table 1, Figures S1 and S2). In step 5, AuNPs were entrapped in 4 followed directly by chelation of Gd into DOTA to make Au-G5-PEG-Alkyne-DOTA-Gd-NHAc, 5. Five equivalents of AuNPs were entrapped in the dendrimer conjugate because initial scouting studies indicated that precipitation occurred in the presence of PBS with the use of more Au, presumably due to the presence of Gd and its counter ions, which is in agreement with the report published by Peng et al. [19]. While purification by ultracentrifugation using water as the washing solvent instead of PBS actually bypassed this problem and enabled the synthesis of higher AuNP equivalent dendrimers, these precipitated during conjugation to Herceptin. Therefore, our studies used 5 and 15 equivalents of Au and Gd respectively. TEM images were acquired to verify the presence of gold (Figure S3). Herceptin-azide, 6, was synthesized by attaching 5-azidopentanoic acid possibly to ~ 40 available lysine side chains [35] on Herceptin. 6 was subsequently clicked to the modified 5 as illustrated in Scheme 1. Herceptin was chosen as the antibody because it has successfully been studied and employed for the treatment of HER-2 positive cancer [30, 31, 40]. Finally, AF647-azide was attached, and also omitted, to synthesize 7 and Au-G5-Gd-Herceptin (7a) respectively. Purity of the final conjugates was verified via UPLC (Figure S4).

### UV-Vis

**Figure 1:**
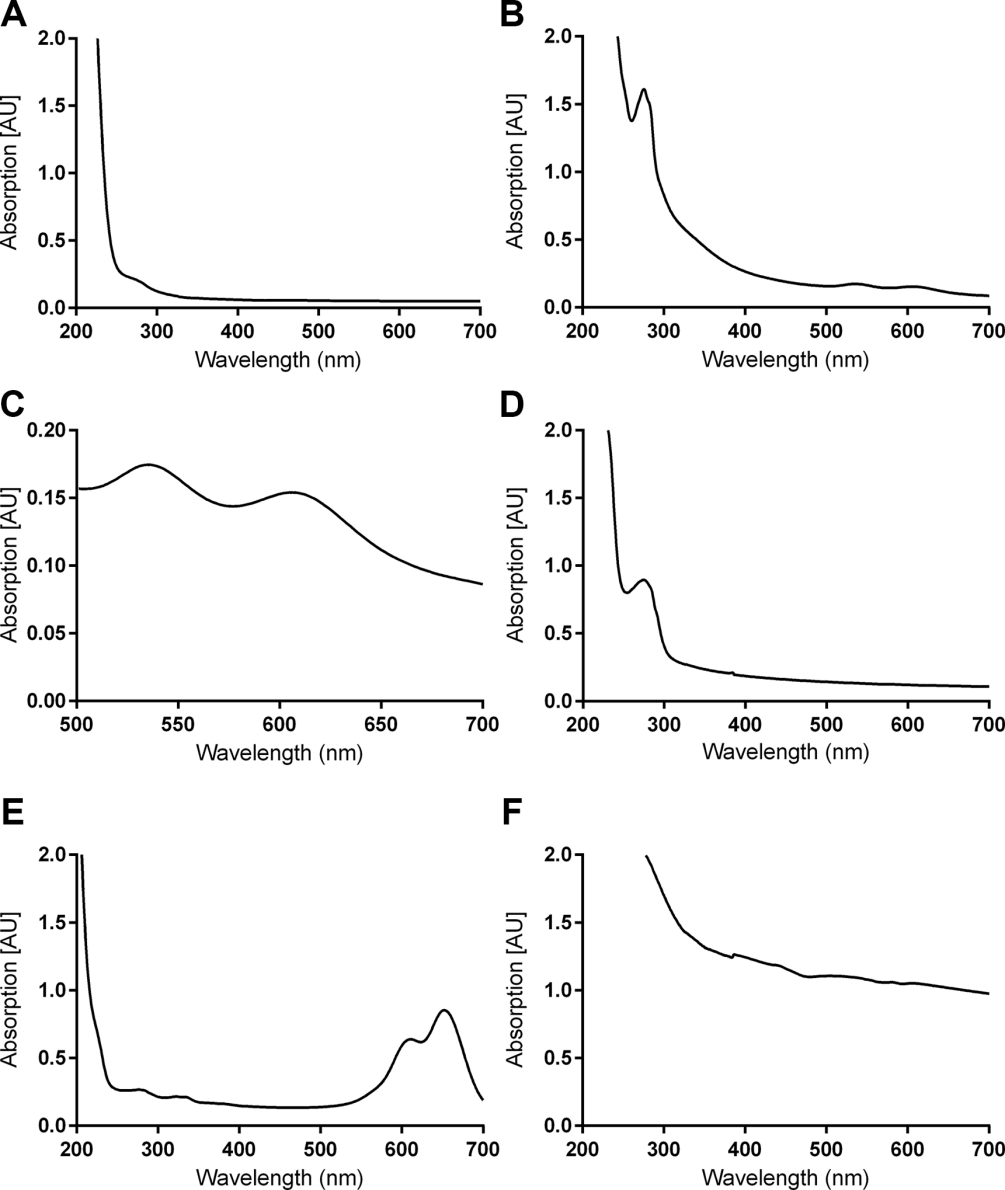
UV-Vis spectra of various compounds Clockwise from the upper right: (**A**) compound 4, (**B**) compound 5, (**C**) the AuNP peak in compound 5, (**D**) compound 6, (**E**) compound 7, (**F**) compound 7a. Note that 7 was measured at 0.1 mg/mL due to the high intensity of AF647. Compounds were disolved in 1 mM HEPES buffer at 1 mg/mL.

Compounds 4, 5, 6, 7, and 7a were examined with UV-Vis (Figure 1) in order to establish the presence of AuNPs and AF647. Peaks around 280 nm and 520 nm in Figure 1 confirm the presence of AuNPs in 5. Figure 1 also confirms the presence of AF647 in 7. Unfortunately, absorption by 5 (Figure 1B) and 6 (Figure 1D) at around 285 nm make confirmation of conjugation to the antibody via UV-Vis impossible.

### Dynamic light scattering (DLS) and zeta potential (ZP)

**Figure 2:**
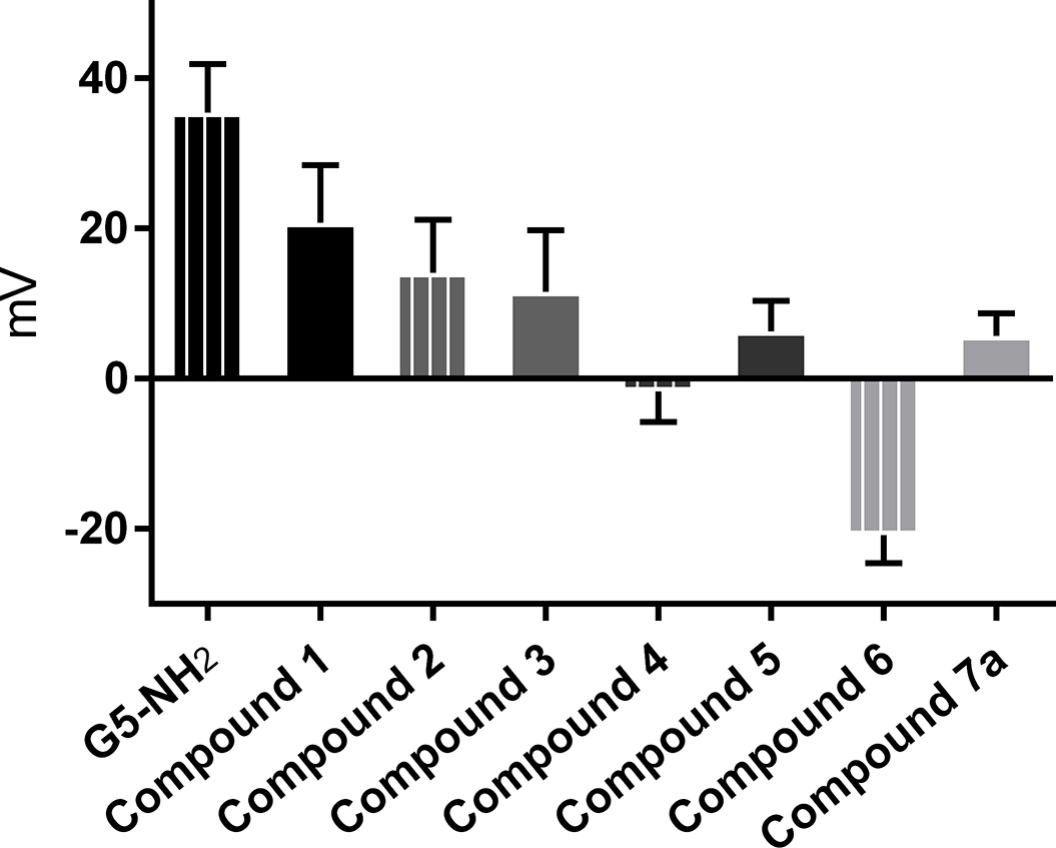
Zeta potential in millivolts of pure and modified dendrimers Error bars denote one standard deviation, *n* = 5. Samples were measured at 1 mg/mL in 1 mM HEPES.

DLS and ZP analyses were performed in order to document the modification steps and to confirm antibody addition to 5. Data are given in Table 2, Figure S5. 5 and 6 each showed a marked increase in size following conjugation. It is important to note that DLS measures the hydrodynamic diameter, which includes hydrating water in the measurement and thus the reported diameter of the compound seems larger than the actual [14]. The ZPs of each conjugation step, measured in order to reflect the decrease in positive charge as amines became amides, are given in Figure 2. Furthermore, the increase in ZP following AuNP entrapment and the ZP increase of 6 after conjugation to 5 further confirm the conjugation between Herceptin and modified G5 dendrimer.

**Table 2:** Z-average dynamic light scattering measurements

Compound	Z-Avg. (d. nm)^a^	PDI^b^
Au-G5-PEG-Alkyne-DOTA-Gd-NHAc (**5**)	28.3 ± 10.2	0.313 ± 0.107
Herceptin-azide (**6**)	154.0 ±7.0	0.509 ± 0.093
Au-G5-Gd-Herceptin (**7a**)	459.0 ± 28	0.471 ± 0.232

a, b ± values denote one standard deviation, *n* = 5.

Note: Z-Avg = Z-Average, PDI = polydispersity index.

### Inductively coupled plasma-optical emission spectroscopy (ICP-OES)

The ratios of Au and Gd in the conjugate were determined by ICP-OES. Conjugate 5 has close to the expected Au and Gd ratios of 4.54 ± 0.58 Au and 11.1 ± 0.5 (reported as average ± standard deviation (SD), sample size *n* = 3). These ratios were calculated by dividing the molar mass of Au and Gd found in each sample by the molar mass of 5 tested in each IPC-OES sample and averaging the results. Interestingly, while according to MALDI-TOF and NMR data the number of DOTA conjugated is less than 11, it was found in previous studies that AuNPs in fact enhance Gd retention beyond that expected with DOTA-Gd chelation alone [25]. We also used a combination of ICP-OES and MALDI-TOF data to estimate the ratio of dendrimer to Herceptin in 7. First, we applied the gold to dendrimer ratio from compound 5 to the amount of Au found in each compound 7 ICP-OES sample; this yielded the moles of dendrimer present. Next, we multiplied the average molecular weight of dendrimer conjugated to AF647 without antibody to find the mass of dendrimer present (55,478.3 g/mol) (Figure S3). The difference after subtracting the dendrimer mass from the sample mass of 7 yielded the Herceptin mass. Comparing the molar equivalents of antibody to dendrimer complex showed on average 5.22 ± 1.1 dendrimers per antibody (one SD, *n* = 3).

### *In vitro* binding and internalization into HER-2 expressing cells

**Figure 3:**
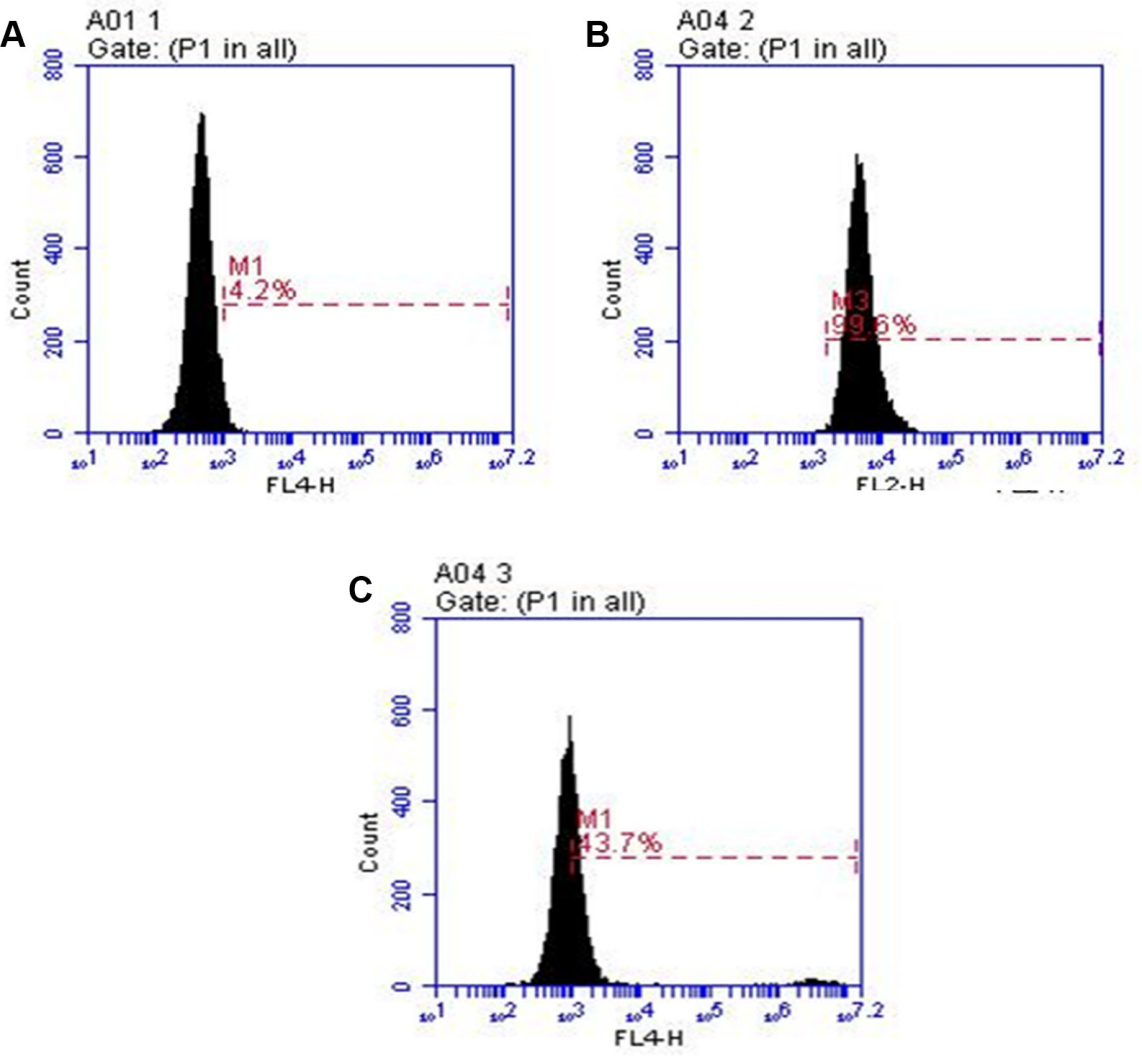
Comparison of binding by 7 to the cells with untreated Herceptin A549 cells were stained with the Herceptin and conjugate 7 and analyzed by flow cytometry. Images: (**A**) shows unstained A549 cells. (**B**) shows pure Herceptin binding. (**C**) shows binding of 7. The cell line was cultured as described in the methods section.

**Figure 4:**
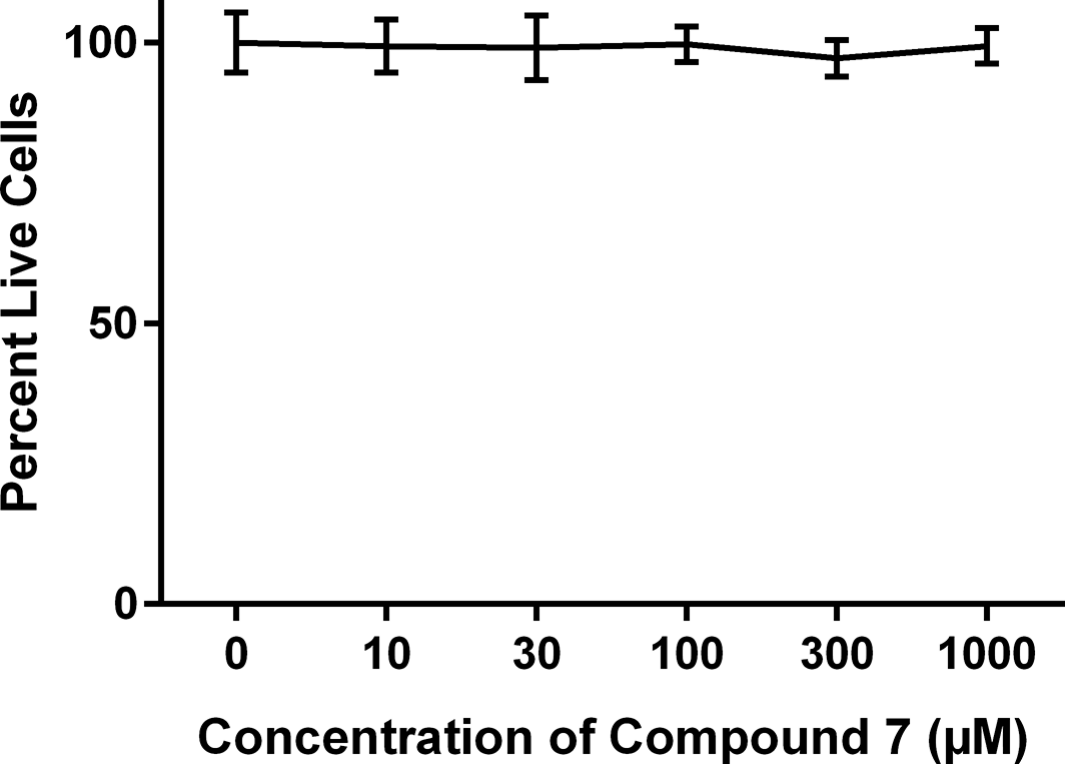
Effect of compound 7 on cell viability A549 cells were incubated at varying concentrations of 7 over 48 hours. The percentage of live cells was analyzed by XTT assay. The PBS control shows 100% of the cells were alive and the conjugate 7 shows 90–95% of cells were alive.

In order to assess the *in vitro* binding capacity of 7 relative to that of Herceptin alone, we used binding and uptake assays. Flow cytometric analysis (Figure 3) shows that twice as many A549 cells were positively stained when incubated with unmodified Herceptin, as compared to A549 cells incubated with compound 7. The discrepancy in the percentage of positively stained cells is likely due to the configuration space of the Herceptin-conjugated dendrimer, 7, as compared to the Herceptin antibody alone. Furthermore, a cytotoxicity assay showed that 7 does not affect A549 cell viability when cells were incubated with the conjugate for time periods up to 48 hours (Figure 4). In addition, the live cell percentage of SKBR-3 was over 95% after 48-hour incubation with 7. This indicates that 7 also had no effect on the SKBR-3 cell viability.

**Figure 5:**
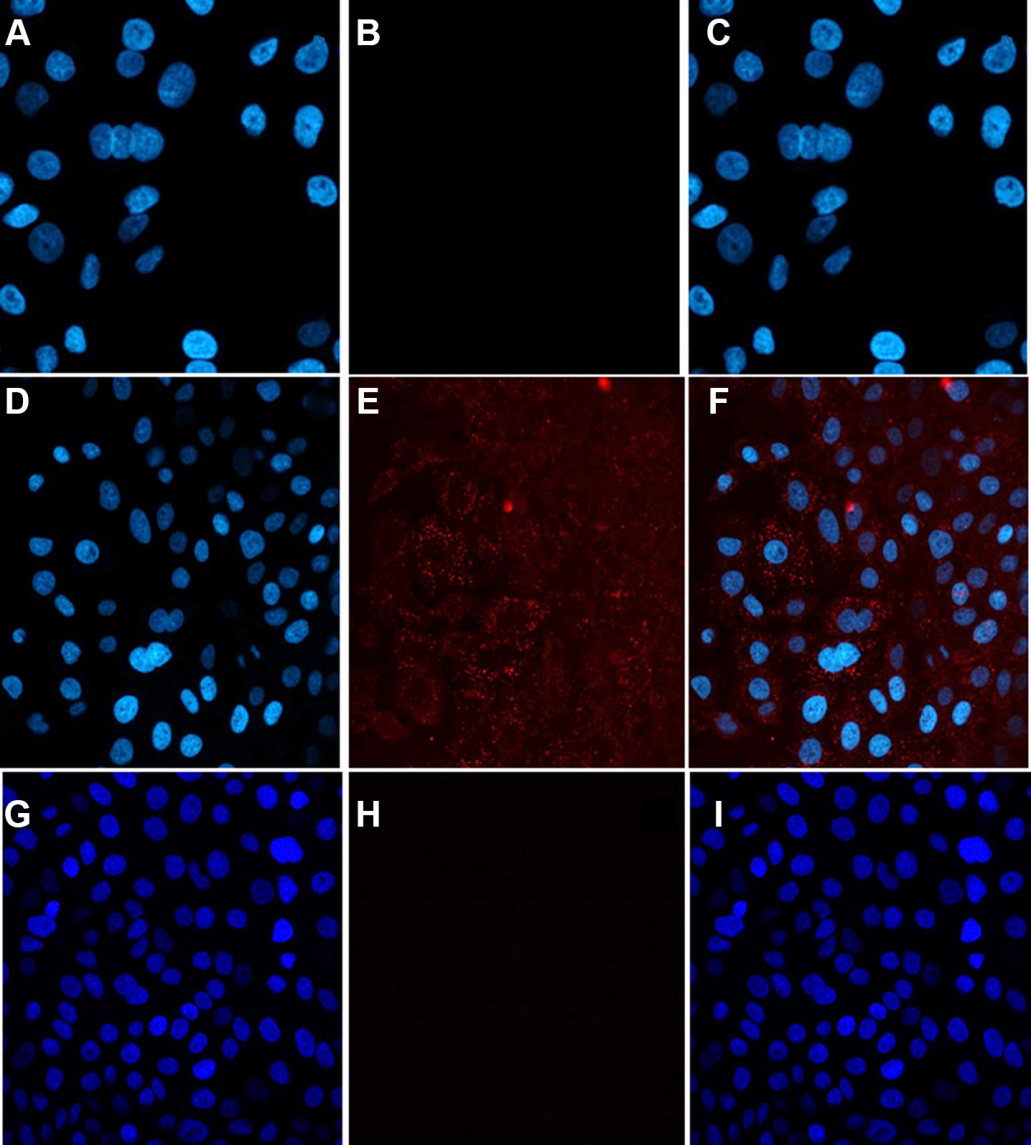
The internalization of compound 7 into A549 cells imaged by confocal microscopy The left panel shows only DAPI staining, the middle panel shows only AF647, and the right panel is an overlay of both. Images (**A–C**) represent PBS control, (**D–F**) show compound 7, and G-I show compound 5. Image F indicates uptake into cells after 18 hours incubation with 7.

**Figure 6:**
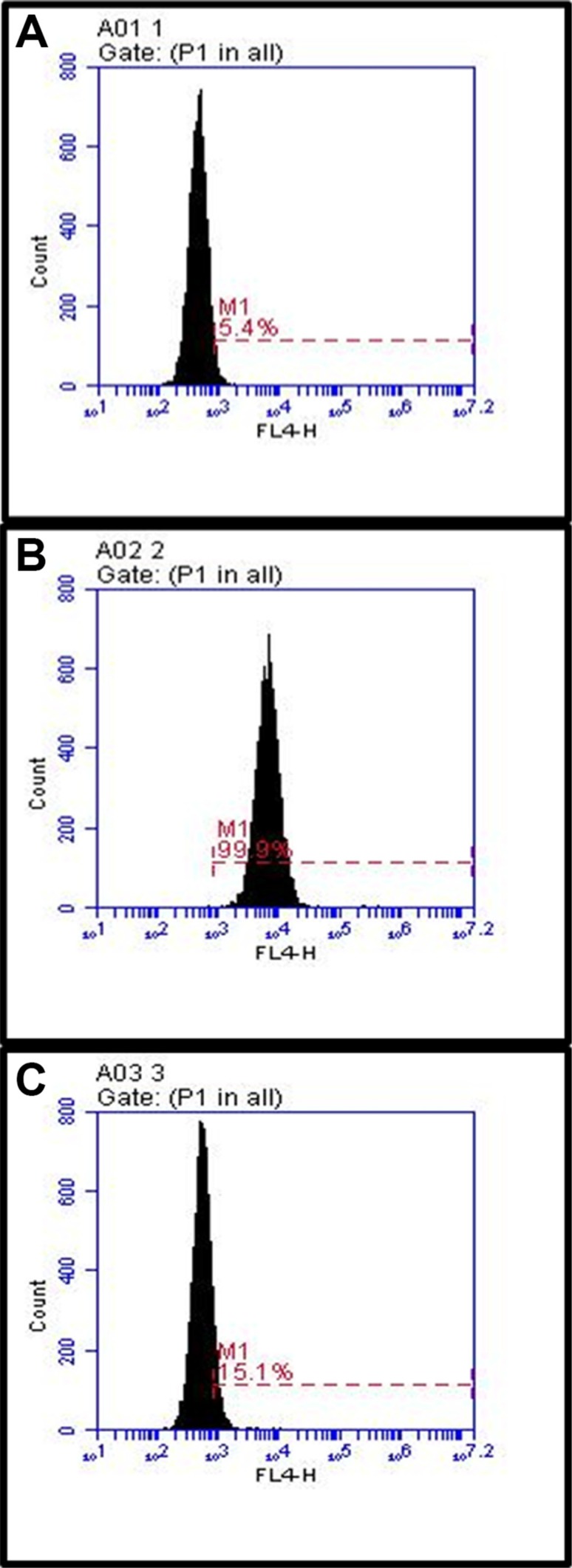
Competition study between Herceptin and compound 7 on SKBR-3 cells The cells were cultured as described in the methods section. Images: (**A**) untreated SKBR-3 cells, (**B**) cells with 7, (**C**) cells incubated with unmodified Herceptin) and then 7. Treatment with Herceptin shows that the dendrimer conjugate exclusively binds to HER-2.

Whether compound 7 can be internalized into A549 cells was determined by a confocal microscopy. Confocal imaging showed that internalization of 7 into A546 cells. However, compound 5 without Herceptin was unable to be internalized into the same cells. This further validated successful production of 7, by conjugation of 5 and 6 (Figure 5). We also noticed a significantly higher cellular uptake and internalization of compound 7 for the 18-hour incubation than the 2-hour incubation, suggesting that a longer incubation period is necessary for the antibody-dendrimer conjugate to fully bind and internalize. Finally, a cellular receptor competition study with 7 was performed with SKBR-3 cells because they exhibit higher levels of HER-2 than A549 cells as shown in Western blot analysis (Figure S6) and thus we envisioned that it would better illuminate any binding discrepancy between Herceptin and the dendrimer antibody complex. The results confirmed the specificity and exclusivity of conjugate 7 against HER-2 overexpressing cells, consistent with the confocal microscopy findings (Figure 6).

The binding activity, internalization and receptor binding competition of 7 altogether demonstrated the specificity of the dendrimer antibody complex. These results suggest that Herceptin activity is retained after conjugation to the dendrimer with imaging agents. Therefore, this conjugate has potential to enhance early detection of breast and small lung cell cancer overexpressing HER-2. With regard to a broader utilization, because click chemistry is applicable to a variety of molecules, other potential targeting agents can be clicked to this Au Gd DENP, which would serve as a standard imaging platform for a variety of cancers.

## MATERIALS AND METHODS

### Materials

All solvents and chemicals were of reagent grade quality, purchased from Sigma-Aldrich (St. Louis, MO), and used without further purification unless otherwise noted. G5 PAMAM dendrimer was purchased from Dendritech (Midland, MI). Monomethyl-PEG_11_-NHS (PEG-NHS) ester was purchased from ChemPep Inc. 3-(4-(2-azidoethoxy)phenyl) propanoic acid was used from previous studies [37]. 2,2′,2′′-(10-(2-((2,5-dioxopyrrolidin-1-yl)oxy)-2-oxoethyl)-1,4,7,10-tetraazacyclododecane- 1,4,7-triyl) triacetic acid (DOTA-NHS) was purchased from Macrocyclics (Dallas, TX). AF647-azide was procured through Life Technologies of Thermo Fisher Scientific (Grand Island, NY). Herceptin was obtained from Genetech (San Francisco, CA) Water used in all experiments was purified using a Milli-Q Plus 185 water purification system (Millipore, Bedford, MA) with resistivity higher than 18 MΩ cm; PBS and Amicon^®^ Ultra-4 centrifugal filter units (MWCO = 10,000 or 100,000) were purchased from Millipore. 35 mm glass bottom culture dishes were purchased from MatTek Corporation, the ProLong Gold Antifade Reagent with DAPI from Life Technologies, and the XTT assay was bought from Roche. DMEM, HEPES buffer and Trypsin-EDTA, all were purchased from Gibco. SKBR-3 cells (HTB-30) and A549 cells (CCL-185) were purchased from American Type Culture Collection.

### Characterization

^1^H NMR spectra were collected on a Varian Inova 500 nuclear magnetic resonance spectrometer. UV-Vis spectra were recorded using a Perkin Elmer Lambda 25 UV-Vis spectrometer (Akron, OH). Analytical ultra-performance liquid chromatography (UPLC) was performed on a Waters Acquity Peptide Mapping System, controlled by Empower 2 software, and outfitted with a photodiode array detector and an Acquity BEH C4 column (100 × 2.1 mm, 1.7 μm) (Milford, MA). The ratios of gadolinium and gold in the conjugates were determined using inductively coupled plasma-optical emission spectroscopy (ICP-OES) on an Optima 2000 DV (Perkin Elmer, Akron, OH). Samples were dissolved in water beforehand and measured in 5% Nitric Acid in water with 0.01% Triton-X 100 using Yttrium (371.029 nm) as an internal standard. Matrix-assisted laser desorption ionization time-of-flight mass spectra (MALDI-TOF-MS) were recorded on a PE Biosystems Voyager System 6050 (Applied Biosystems, Grand Island, NY). 1 mg/ml of matrix in acetone/methanol was first spotted, followed by a 0.1 mg/mL of sample in a 50% matrix solution (2,5-dihydroxybenzoic acid, 10 mg/mL). TEM images were recorded on a JEOL 2100F and handled using ImageJ software (JEOL, Freising, Germany). For dynamic light scattering analysis and zeta potential a Malvern Zetasizer Nano-ZS was used (Worcestershire, UK). Samples were 1 mg/mL in 1 mM HEPES buffer at pH 7, and zeta potentials were calculated using the Smoluchowsky model. All other sample preparations followed previous methods [31, 34, 37].

### Synthesis of G5-PEG-Alkyne-DOTA-NHAc

The conjugate G5-PEG-Alkyne-DOTA-NHAc (4) was synthesized following published procedures with some modifications [2, 19, 25, 37, 38]. First, 40 equivalents of trimethylamine (TEA) (64 mg, 0.63 mmol) were added to G5-NH_2_ (418 mg, 0.016 mmol) in deionized water (DI) water (8 mL) and set stirring. Then, 25 equivalents of PEG-NHS (272 mg, 0.397 mmol) dissolved in methanol (2.5 mL) were then added dropwise over 10 minutes to the dendrimer solution. The reaction mixture was stirred for 2 days and the product mixture was purified via ultrafiltration five times in phosphate buffered saline (PBS), and 5 times in DI water using a 10,000 MWCO Amicon centrifuge device to isolate G5-PEG-NH_2_ (1) [37]. Purification of reaction mixtures to isolate pure desired products was performed following this procedure (washing with PBS X 5 and DI water X 5) unless otherwise noted. Next, twenty equivalents of alkyne linker (20.4 mg, 0.0999 mmol), with one time excess EDC (38.3 mg, 0.200 mmol) and NHS (23.0 mg, 0.2 mmol) in 2 mL of acetonitrile (ACN). After 4 hours, the solution was added dropwise into an aqueous solution (2 mL) containing 1 (200 mg, 0.005 mmol). The final solution stirred overnight and the purified product G5-PEG-Alkyne-NH_2_ (2) was recovered. Then, conjugate 2 (180 mg, 0.004 mmol) and 20 equivalents of TEA (8.5 mg, 0.008 mmol) were dissolved in DI water (3 mL) and set stirring. Immediately after, 15 equivalents of DOTA-NHS (47.8 mg, 0.063 mmol) in DI water (1 mL) were added dropwise and the reaction mixture was allowed to stir overnight. Purification yielded G5-PEG-Alkyne-DOTA-NH_2_ (3). Following this, compound 3 (165 mg, 0.003 mmol) was stirred with TEA (100.2 mg, 0.990 mmol) in 4 mL anhydrous methanol and capped using a solution of 200 equivalents of acetic acid (67.4 mg, 0.660 mmol) in methanol (2 mL, anhydrous) [7, 39]. Purification followed to afford compound 4.

### Synthesis of Au-G5-PEG-Alkyne-DOTA-Gd- NHAc

Au DENPs with gadolinium were made using previously defined methods [26, 40] but with some adjustments. An aqueous solution (8 mL) of 4 (40 mg, 0.0007 mmol) was given 5 molar equivalents of HAuCl_4_ (1.26 mg, 0.0037 mmol) in DI water (0.126 mL); the reaction mixture was stirred for 30 minutes. Next, an ice cold, 0.07 mL NaBH_4_solution (0.70 mg, 0.019 mmol) in 5 times molar excess to HAuCl_4_ was added dropwise. After 4 hours, an aqueous solution (0.334 mL) of Gd(NO_3_)_3_ (33.4 mg, 0.074 mmol) was added. After overnight stirring, the product Au-G5-PEG-Alkyne-DOTA-Gd-NHAc, 5, was isolated after centrifuging the product mixture five times in DI water.

### Synthesis of Herceptin-azide

5-azidopentatonic acid (1.05 mg, 0.007 mmol) reacted with EDC (26.8 mg, 0.140 mmol) and NHS (16.1 mg, 0.140 mmol) in ACN (0.2 mL). After 4 hours, Herceptin (20 mg, 1.36 μmol) was dissolved in water along with 60 eq. of TEA (0.83 mg, 0.008 mmol), followed by ACN (10 μL) and stirred at room temperature. Five minutes later, 40 eq. of 5-azidopentanoic acid from the initial solution (0.0158 mL) were added to the Herceptin solution and left to stir over night. Purification was conducted with 100,000 MWCO centrifugal devices, 5 times in PBS and DI water each, then lyophilized to yield 6 as a white solid (39%, 10.41 mg).

### Synthesis of Au-G5-Gd-Herceptin-AF647

Compound 6 (1.77 mg, 11.8 μmol) and 6.8 molar equivalents of 5 (4.28 mg, 0.080 μmol) were combined and dissolved in CuSO_4_ (0.1 mL, 1 mg/mL) and sodium L-ascorbate (Na Asc) (0.4 mL, 1 mg/mL), and conjugated using copper catalyzed click reaction [34]. After stirring for an hour, 37 μL of AF647 (assumed M.W. 1200 g/mol) in DMSO (0.5 mg/100uL) were added and the reaction left overnight. The reaction mixture was purified using 100,000 MWCO centrifugal filtration devices. Purification consisted of 5 cycles (20 min at 4800 rpm) using DI water. The purified sample was lyophilized to yield the dendrimer Herceptin conjugate, 7, as a blue solid (92%, 4.75 mg). Half of the reaction did not receive AF647 in order to allow for zeta potential and DLS measurements (7a).

### Cell cultures

A549 human lung carcinoma cells lines were grown in RPMI 1640 medium supplemented with 10% fetal bovine serum (FBS) and 1% penicillin-streptomycin at 37°C with 5% CO_2_. SKBR-3 human breast adenocarcinoma cell lines were cultured in Dulbecco's Modified Eagle Medium (DMEM) under the same conditions as mentioned above. All cell lines were grown in monolayer to approximately 80% confluence before being split.

### Cell viability

Cell viability of A549 and SKBR-3 cells in the presence of conjugate 7 was assessed using an XTT assay. A549 or SKBR-3 cells were cultured in a 96-well tissue culture plate (12–15,000 cells/per well) with complete RPMI 1640 medium and incubated in 37°C with 5% CO_2_ until 50–60% confluent. Conjugate 7 was added at 57 μg/μL, and cells were incubated for 48 hours. Directly prior to analysis, 50 μL of XTT reagent (50 parts of Labeling Reagent to 1 part electron-coupling reagent) were added to 100 μL PBS in each well. Spectra-photometrical absorbance was measured as Delta OD 492–690 nm in order to calculate the percentage of live cells.

### Flow cytometry

The cellular binding of conjugate 7 to cell surface HER-2 was ascertained by flow cytometry. The unmodified Herceptin was used as a control. Upon reaching 80% confluence in 96 well plates, A549 cells were briefly digested with 0.25% Trypsin-EDTA, then recovered with complete RPMI 1640 medium. Before staining, all cells were blocked with 10% FBS in PBS for 30 minutes at 4°C and washed one time with 0.1% bovine serum albumin (BSA) in PBS. Control cells were incubated with 3 μL (per 0.5 × 10^6^ cells) of unmodified Herceptin (10 μg/μL), and sample cells were incubated in 0.57 μg/μL of 7 (per 0.5 × 10^6^ cells); both had 0.1 mL of 0.5% BSA/PBS. Ambient conditions were the same as blocking. After 30 minutes at 4°C, each sample was washed three times with 1 mL of 0.1% BSA/PBS and centrifuged for 5 minutes. Samples incubated with conjugate 7 were resuspended in 0.5 mL of 0.1% BSA/PBS and placed on ice until analysis. To the control cells, 20 μL (per 1 × 10^6^ cells) of PE-Ig, k Light Chain secondary antibody were added and incubated under blocking conditions. The sample was washed three times using 1 mL of 0.1% BSA/PBS and resuspended in 0.5 mL of 0.1% BSA/PBS. The samples were analyzed with an Accuri C6 Flow Cytometer (BD Biosciences, San Jose, CA).

### Confocal imaging

0.5 × 10^6^ A549 cells were plated onto cover slips and placed in 35 mm glass bottom culture dishes. 24 hours later the cells were incubated with conjugate 7 or 5 for 2 hours or 18 hours. Cells were then rinsed twice with PBS and fixed with 2% paraformaldehyde (1 mL) in PBS for 15 minutes. Cells were rinsed twice with PBS and mounted with ProLong Gold Antifade Reagent with DAPI. The dishes were stored at 4°C until analysis using an Olympus FluoView 500 Laser Scanning Confocal Microscope (Olympus, Waltham, MA).

### Competition of Au-G5-Gd-Herceptin-AF647 with Herceptin

SKBR-3 cells were digested with 0.25% Trypsin-EDTA and recovered with DMEM complete medium. Cells were blocked with 10% FBS in PBS for 30 minutes at 4°C and washed once with 0.1% BSA in PBS. 30 μL of unmodified Herceptin (10 μg/μL) were added to the cells and incubated for 30 minutes at 4°C. The samples were washed twice with 0.1% BSA in PBS. Cells then were incubated with 7 (0.57 mg/mL). After 30 minutes at 4°C, the cells were washed twice with 0.1% BSA in PBS and resuspended in 0.5 mL 0.1% BSA in PBS. The cells were analyzed for inhibition via flow cytometry.

## CONCLUSIONS

This study is the first demonstration of a versatile PAMAM dendrimer conjugate that consists of AuNPs and Gd as CT/MRI dual modus imaging agents as well as the monoclonal antibody Herceptin. Additionally, Herceptin has been conjugated to the modified dendrimer via the Cu catalyzed click reaction, demonstrating the successful application of the click reaction in conjugating two macromolecules. UV-Vis and ICP-OES confirm encapsulation of AuNP and Gd. A DLS study clearly indicated an increase in particle size of modified dendrimer antibody conjugate from modified dendrimer 5 and Herceptin-azide 6, which confirms the formation of the dendrimer antibody conjugate. In cell culture studies, we observed exclusive *in vitro* internalization into HER- 2 positive cells by this conjugate. In addition, conjugate 7 caused no cytotoxicity when internalized, nor when incubated with cells exhibiting normal HER-2 expression. Altogether, the AuNP and Gd bearing dendrimer-antibody complex provides an improved model for the conjugation and delivery of not only imaging modalities but also cytotoxic drugs or siRNA. This novel targeted compound stands as a potential new clinical approach for the early detection of HER-2-positive cancers.

## SUPPLEMENTARY MATERIALS


