# Supplementary material for: Dendrimer antibody conjugate to target and image HER-2 overexpressing cancer cells
Source: Oncotarget. 2016 Apr 28;7(24):36002–13. doi: 10.18632/oncotarget.9081 (PMC5094978; doi:10.18632/oncotarget.9081)
Supplement: Supplementary file 1 [file oncotarget-07-36002-s001.pdf]

Dendrimer antibody conjugate to target and image HER-2 overexpressing cancer cells

Supplementary Materials

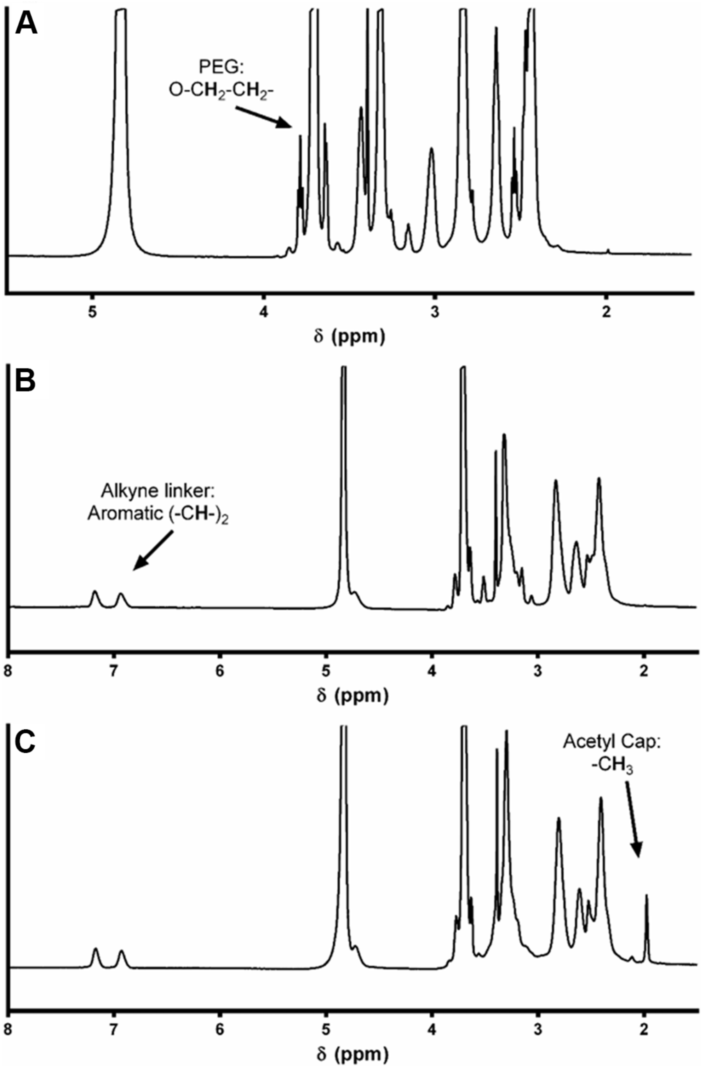

Supplementary Figure S1: NMR shifts for the initial dendrimer modification steps. (**A**) compound 1, (**B**) compound 2, (**C**) compound 4. Indicatory peaks of the respective modifications are labeled on each sub-figure. Samples were measured in 2 mg/mL D_2O.

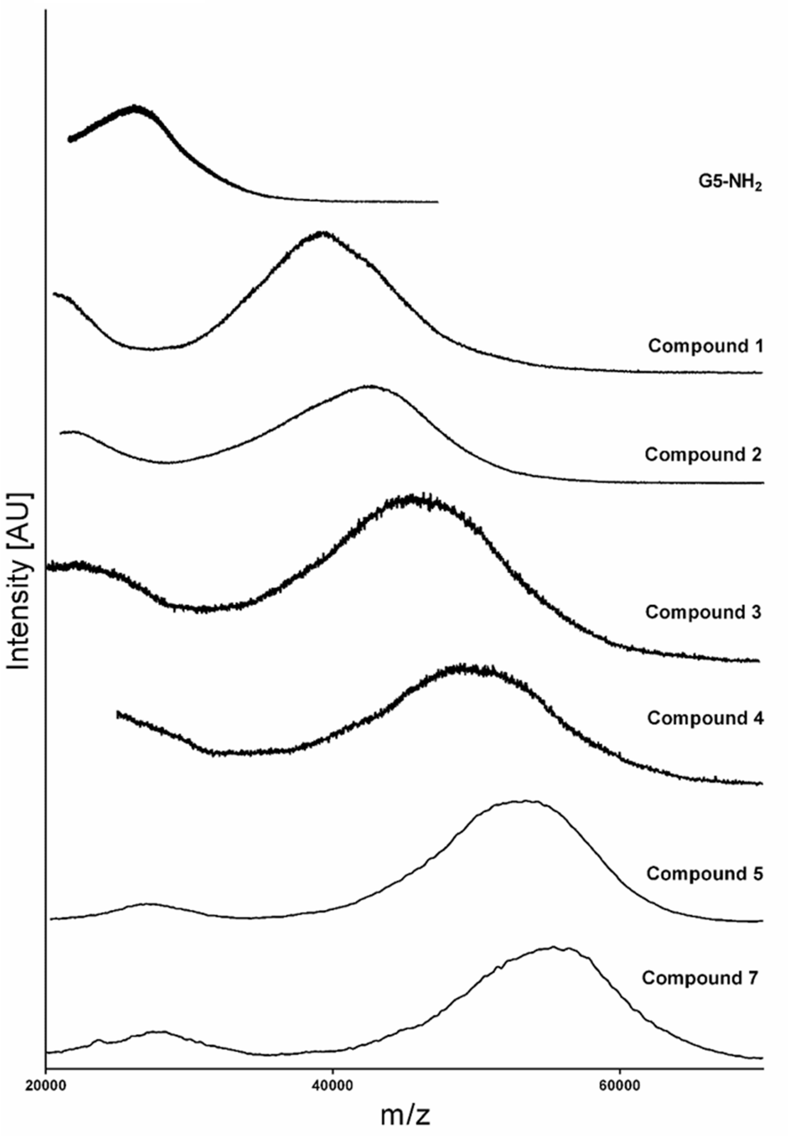

Supplementary Figure S2: Depicted are the MALDI-TOF spectra for each compound. The plot of 7 displays only the dendrimer's mass.

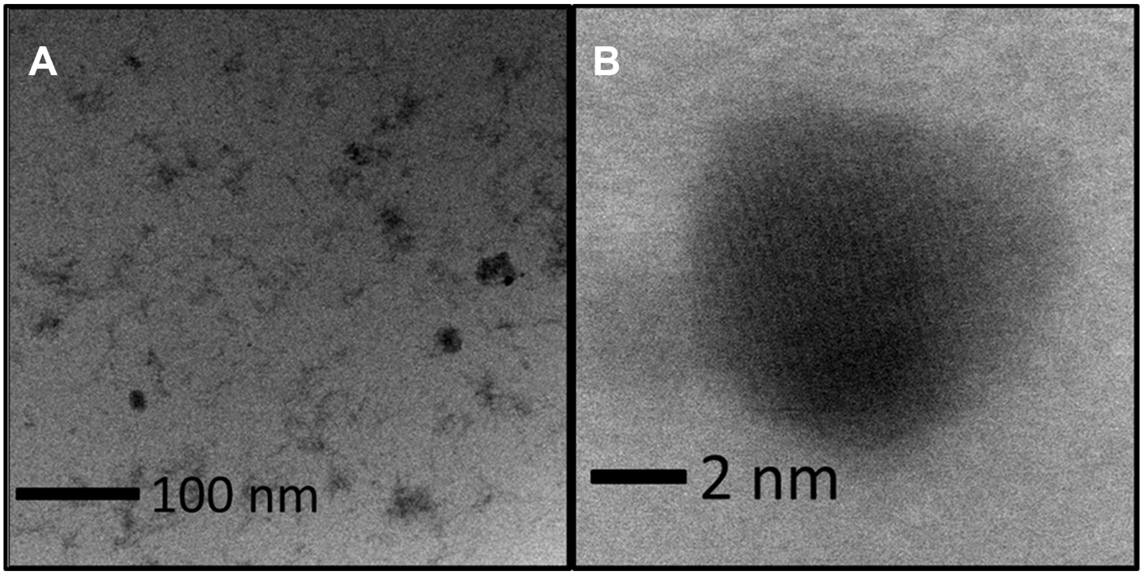

Supplementary Figure S3: TEM image of the dendrimer Herceptin conjugate. Image (**A**) depicts the dendrimer Herceptin complex with gadolinium and gold nanoparticles. Image (**B**) shows an AuNP at roughly five nanometers.

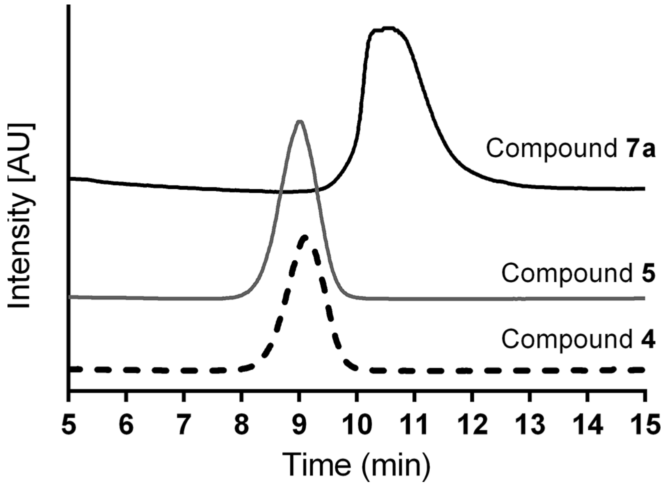

Supplementary Figure S4: UPLC Chromatograms of various compounds. (**A**) shows pure Herceptin, (**B**) shows 4, 5, and 7. Samples were dissolved in DI water and measured at 285 nm.

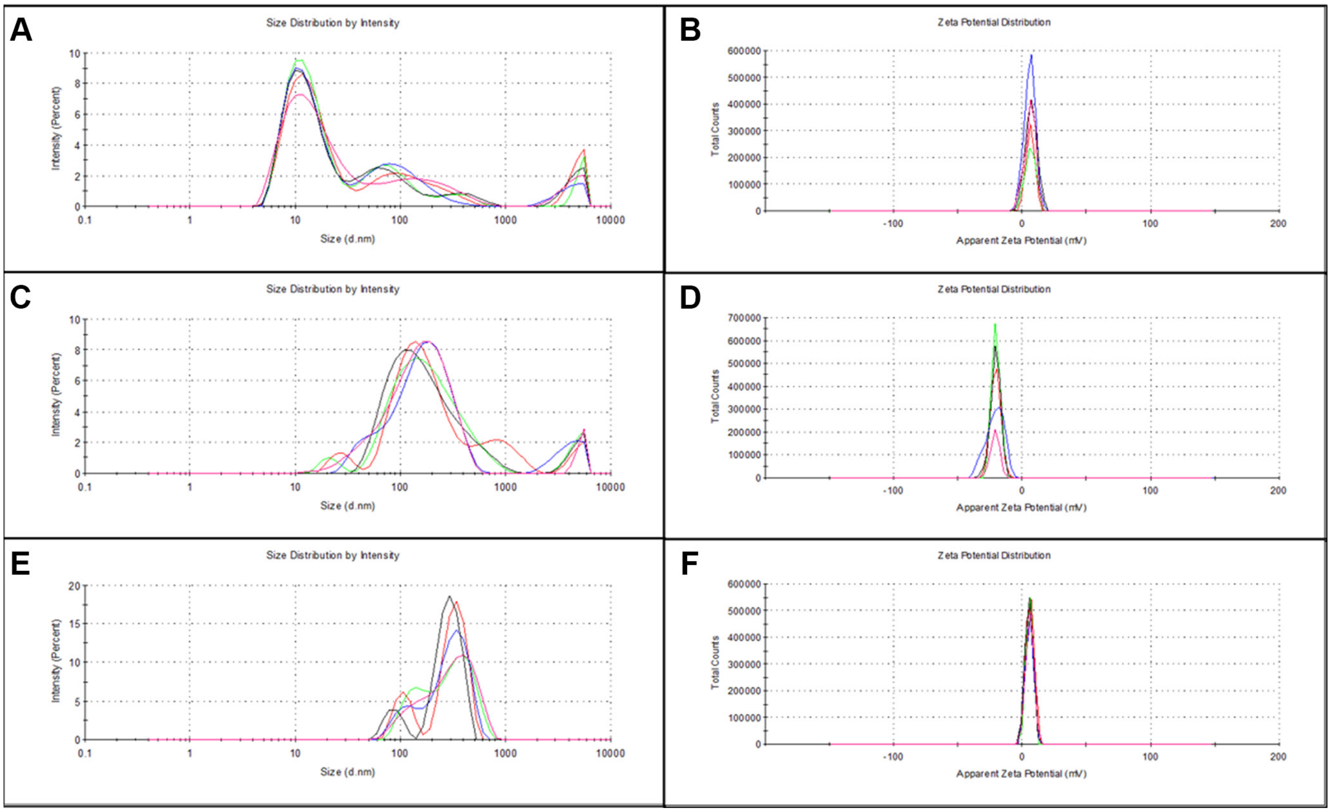

Supplementary Figure S5: (**A**) DLS plot of compound Au-G5-PEG-Alkyne-DOTA-Gd-NHAc (5) (28.3 ± 10.2 d. nm), (**B**) Zeta potential plot of compound 5 (6.3 ± 4.1 mV), (**C**) DLS plot of compound Herceptin-azide (6) (154.0 ± 7.0 d. nm), (**D**) Zeta potential plot of compound 6 (–20.86 ± 3.72 mV), E) DLS plot of Au-G5-Gd-Herceptin (7a) (459.0 ± 28 d. nm), F) Zeta potential plot of compound 7a (5.72 ± 3.0 mV). Samples were measured in 1mM HEPES buffer at 1mg/mL (reported as mean ± one SD, *n* = 5).

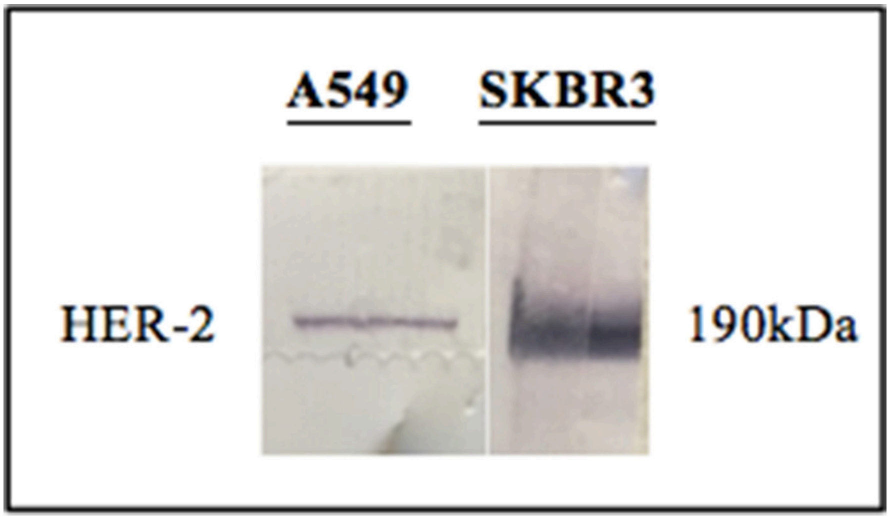

Supplementary Figure S6: Western blot analysis of A549 and SKBR-3 cell lines. 15 µg of total protein was loaded into each lane. The protein concentration was determined by BCA assay. The left lane: cell lysate of A549 cells; the right lane; cell lysate of SKBR-3 cells.
